# The Dartmouth Falls Prevention Training Program: Primary Care and Community-Based Organization Collaboration

**DOI:** 10.1093/geroni/igab046.1422

**Published:** 2021-12-17

**Authors:** Dawna Pidgeon

**Affiliations:** Dartmouth Centers for Health and Aging, Lebanon, New Hampshire, United States

## Abstract

Falls are a leading cause of fatal and non-fatal injuries in older adults. Older adult participation in community-based falls prevention programs can significantly reduce falls risk, however, identifying and referring individuals to appropriate programs can be challenging. Through Administration for Community Living (ACL) funding, we have developed a comprehensive Dartmouth Falls Prevention Training Program for healthcare and community based organizations that includes (1) Falls screening in primary care; (2) “Balance Days”, a community-based education and balance screening event encompassing falls risk stratification and coaching into programs; (3) Instructor Training for Tai Ji Quan: Moving for Better Balance®, a highly effective falls prevention program; and (4) Implementation Training, a research informed workshop shown to enhance community-based program sustainability through participant retention. We will share strategies for sustainable collaborations between primary care and CBOs to reach at-risk individuals and improve lives and decrease costs associated with falls.

